# Real-World Data and Real-World Evidence in Healthcare in the United States and Europe Union

**DOI:** 10.3390/bioengineering11080784

**Published:** 2024-08-02

**Authors:** Kelly H. Zou, Marc L. Berger

**Affiliations:** 1 Viatris Inc., Canonsburg, PA 15317, USA; 2 AI4Purpose Inc., New York, NY 10016, USA; 3 Marc L. Berger, LLC., New York, NY 10026, USA; mlberger301@gmail.com

**Keywords:** real-world data, real-world evidence, data types, data discoverability, data quality, data privacy, interoperability

## Abstract

The use of real-world data (RWD) for healthcare decision-making is complicated by concerns regarding whether RWD is fit-for-purpose or is of sufficient validity to support the creation of credible RWE. An efficient mechanism for screening the quality of RWD is needed as regulatory agencies begin to use real-world evidence (RWE) to inform decisions about treatment effectiveness and safety. First, we provide an overview of RWD and RWE. Data quality frameworks (DQFs) in the US and EU were examined, including their dimensions and subdimensions. There is some convergence of the conceptual DQFs on specific assessment criteria. Second, we describe a list of screening criteria for assessing the quality of RWD sources. The curation and analysis of RWD will continue to evolve in light of developments in digital health and artificial intelligence (AI). In conclusion, this paper provides a perspective on the utilization of RWD and RWE in healthcare decision-making. It covers the types and uses of RWD, data quality frameworks (DQFs), regulatory landscapes, and the potential impact of RWE, as well as the challenges and opportunities for the greater leveraging of RWD to create credible RWE.

## 1. Introduction

The healthcare landscape is rapidly shifting toward the utilization of real-world data (RWD) and real-world evidence (RWE) to inform decision-making processes. Unlike traditional clinical trials that operate within controlled environments, RWD and RWE offer insights derived from real-life patient experiences in diverse settings. However, the journey toward leveraging the full potential of RWD and RWE can only be maximized by overcoming several challenges ranging from data discoverability, transparency in data curation and data quality assurance, the linkage of data across various platforms, and the protection of sensitive data [1,2,3,4,5,6,7,8,9,10].

“Real-world data [FDA] are data relating to patient health status and/or the delivery of health care routinely collected from a variety of sources. Examples of RWD include data derived from electronic health records, medical claims data, data from product or disease registries, and data gathered from other sources (such as digital health technologies) that can provide information regarding patient health status.”

United States (US) Food and Drug Administration (FDA) [5]

Consequently, “Real-world evidence [RWE] is the clinical evidence about the usage and potential benefits or risks of a medical product derived from analysis of RWD”. Together, they offer a glimpse into how treatments and interventions perform in diverse real-world settings beyond traditional clinical trials (CTs).

One of the primary advantages of RWD and RWE lies in their ability to capture the complexities of patient care as it happens in the real world. Other than pragmatic clinical trials (PCTs), most regulatory-purposed CTs involve strict inclusion criteria and controlled environments that do not reflect the diversity of patient populations, treatment patterns, and healthcare delivery systems.

The promise of RWD and RWE lies in their potential to enhance the development of new therapeutic strategies and support the creation of a learning healthcare system that results in continuous quality improvement from the merging of clinical research and healthcare delivery. Observational data have long been utilized by regulators to identify significant adverse events associated with treatments; however, both in the US and Europe, regulators are exploring how to appropriately use RWE derived from observational studies to inform their decisions about treatment effectiveness. In addition, good-quality observational RWD enables the spectrum of healthcare stakeholders to obtain a more comprehensive view of patient health and treatment outcomes, facilitating the identification of trends, patterns, and insights that may have otherwise gone unnoticed.

This article provides a perspective on the utilization of real-world data (RWD) and real-world evidence (RWE) in healthcare decision-making. It covers the importance of RWD/RWE, data quality frameworks (DQFs), regulatory landscapes, and the potential impact of RWD, as well as the challenges and opportunities for appropriately expanding the use of RWD to create credible RWE.

## 2. RWD Types and Use

The analysis of RWD for most of the last four decades has focused on structured data that has been coded in a standardized fashion. This includes diagnosis codes, procedure codes, prescriptions written, dates of eligibility, dates of service, and many others. Most of this information was obtained from administrative claims and pharmacy records. The strength of this data is that it is very complete. The limitations of these data are that coding is not always accurate and that exposure and outcomes of treatments must be inferred. Recent initiatives have focused on transparency in the curation procedures utilized to acquire and transform “raw” coded RWD into “analyzable” RWD to determine the quality of the RWD and whether it is fit-for-purpose to answer specific scientific questions. These issues will be explored later in this paper. Interventional studies involving animals or humans and other studies that require ethical approval must list the authority that provided approval and the corresponding ethical approval code.

Since the 21st Century Cures Act, when RWE was defined by the U.S. Congress [7], many new sources of RWD have become available, including electronic health records (EHRs), ‘omics data, specimens, voice recordings, images, texts, and sensor data from wearable devices and health apps. While a minority of data in EHRs are recorded in structured fields, most of the data are not, which may only be accessible as free-text or reports in portable document formats (PDFs).

**Figure 1:**
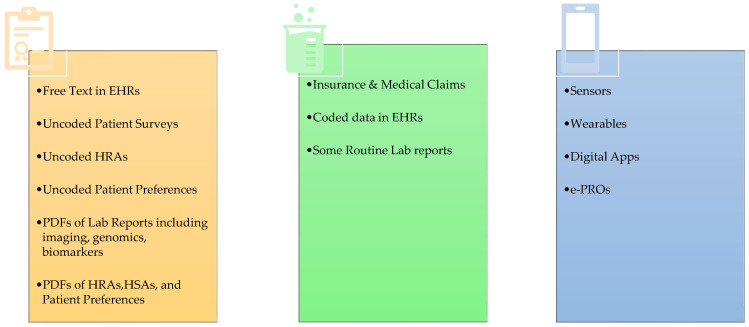
Types of RWD (EHR = Electronic Health Record; HRA = Health Risk Assessment; HSA = Health Status Assessment; PDF = Portable Document Format; PRO = Patient-Reported Outcome; RWD = Real-World Data).

The breadth and depth of this data are tremendous; however, the curation of this data into analyzable data sets requires significant effort. Free-text doctor notes can be converted into structured, coded data manually or through natural language processing and machine learning; however, while expert manual coding of data is well accepted, these other methods have not yet been validated. Hence, their acceptance by regulatory authorities has been limited. For other sources of data, such as wearable sensors, there has not been sufficient transparency in the disclosure of how data has been processed or demonstration of the clinical significance of their findings. The sources of RWE continue to expand, particularly with emerging digital sources (Figure 1). The greater variety and availability of RWD are revolutionizing research and development, patient care, and the work of regulatory and health technology assessment (HTA) agencies.

The findability, accessibility, interoperability, and reusability (FAIR) principles outline the dimensions of RWD that are fundamental considerations in assessing their usefulness [8]. Such data must also be of sufficient quality and fit-for-purpose. Research purpose-driven prospective collection of RWD has advantages over RWD collected for non-research purposes in that it ensures the collection of critical data elements and often includes ongoing data quality checks. These are common characteristics of PCTs, prospective observational studies, and registries. The disadvantage of these approaches is that they may take a long time to complete and require a much greater assignment of effort and funding to complete in comparison with the secondary use of RWD collected during healthcare delivery.

Secondary use of routinely collected RWD generally requires less effort but faces its own challenges with respect to transparency of how the research is conducted and, therefore, its credibility. It has been recommended that such research be conducted transparently, including public posting of the protocol and hypotheses tested prior to analyzing the RWD. Furthermore, RWD scientists describe what efforts were employed in selecting an RWD source to use (such as numbers of patients and events) and reporting any changes to the protocol and their rationale that occur while the RWD is analyzed. In the absence of randomization, the imputation of causality is more difficult due to issues of bias and confounding. These will be discussed in more detail later in this paper. For example, the criteria for designing RWD studies are summarized in Table 1.

Data discoverability and linkage remain significant hurdles, with RWD often scattered across disparate systems and sources. Addressing these challenges requires concerted efforts from stakeholders across the healthcare ecosystem. Initiatives aimed at improving data standardization, interoperability, and privacy protection are essential. Collaboration between healthcare providers, technology companies, regulators, and patients themselves is key to building a robust infrastructure for RWD and RWE utilization.

One useful tool for data linkage is tokenization [9,10]. This process converts patient identifiers into codes or tokens that allow researchers to connect patient data from various sources without the need to know the identity of the patients. Implementing tokenization early in a study may enable researchers to use existing data sources while reducing the need for new data collection. Making tokens involves straightforward steps, but it is crucial to consider factors like defining research questions, obtaining consent, and training staff on using tokens. Tokenization is typically performed using cloud-based data systems.

## 3. Acceptability of RWD for Regulatory Decision-Making

In the US, RWD/RWE initiatives are driven by various regulatory frameworks, such as the FDA’s RWD and RWE guidance documents [5,11,12,13,14,15,16,17,18,19]. The FDA’s “Advancing Real-World Evidence Program” [19] follows various regulatory frameworks. The EU has embarked on similar initiatives and supported the development of systems to facilitate data sharing while ensuring compliance with privacy regulations. The European Health Data Space (EHDS) aims to foster cross-border data exchanges and transfers to support healthcare delivery, research, and innovation. The EHDS seeks to strike a balance between enabling data sharing for legitimate purposes and safeguarding individual privacy rights [20]. Recently, the EMA has written a reflection paper [21]. Ultimately, regulators want to be convinced that the RWD used to generate RWE is reliable, relevant, and fit-for-purpose (Table 2). The definitions of these terms, although they overlap, are not identical across jurisdictions; this represents an opportunity for harmonization efforts in the future. Regulators also want to be assured that non-interventional RWD studies are designed and conducted rigorously. With respect to the latter issue, a consensus on good practice recommendations has yet to emerge. By implementing robust data governance frameworks and fostering collaboration between stakeholders, healthcare organizations can harness the power of real-world data while safeguarding patient privacy and complying with regulatory requirements.

At its base, the creation of RWE requires access to RWD sources that are reliable, relevant, and fit-for-purpose. Deciding whether an RWD source is fit-for-purpose depends on the assessment of reliability and relevance in the context of the regulatory issue being considered. Reliability and relevance are the overarching framework for the assessment of data quality. The data quality framework in the EU is more detailed and encompasses various dimensions as compared to the US. [22,23] (Table 2). For the EMA, the major considerations are transparency, reliability, extensiveness, coherence, and timeliness. Twelve DQ dimensions were identified in a systematic literature review, and it was concluded that while there was much overlap across the proposed DQF’s, the definitions of “dimensions” were quite variable [24].

To provide assistance to researchers and sponsors facing this current complexity, we have recently developed a research-intuitive set of screening criteria to assess whether potential RWD sources for planned research studies are fit-for-purpose and of sufficient quality to support the creation of trustworthy RWD (Table 1) [25]. We have given this tool the acronym ATRAcTR (meaning: Authentic Transparent Relevant Accurate Track-Record).

**Table 1:** ATRAcTR data quality criteria.

Dimensions	Concepts
Authenticity	Provenance documentation: what, why, who, how, and when data was collected Ability to verify provenance through data access Changes in data collection over time De-identification and privacy protection
Transparency	Data Extraction, Transformation, and Loading Handling of incorrect or missing data Data linkage Handling of free-text Handling of changes in coding conventions Data latency and refresh rate Inclusion of synthetic data
Relevancy	Sample size of the population of interest Representativeness Length of follow-up Demographic characteristics
Accuracy	Data quality (integrity) checks, including conformance, plausibility, completeness, uniqueness of patient records, and continuity of data collection Ability to audit key variables
Track Record	Historical performance of data set in creating credible, real-world evidence

These criteria are consistent with the frameworks promoted by the FDA and the EMA within a simplified framework and employ terminology directly relatable to the work performed by clinical researchers (Table 2).

**Table 2:** A comparison of multiple DQFs.

FDA		EMA		ATRAcTR
Data Reliability	Accuracy Completeness Provenance Traceability	Data Reliability	Precision Accuracy Plausibility	Data Authenticity Data Transparency Data Accuracy
		Data Extensiveness	Completeness Coverage	
		Data Coherence	Format Structural Semantic Uniqueness Conformance Validity	
		Data Timeliness		
Data Relevance	Exposure Outcomes Adequate Sample size	Data Relevance		Data Relevance
Study Design	Employ Causal Inference Framework			
				Data Track Record

Beyond data quality, it has become apparent that study design is equally, if not more, important to the creation of credible RWE. Much of this work has demonstrated that discrepancies between RCTs and RWD studies that attempted to emulate their findings were due to two factors: not embedding the study design within a causal incidence framework or not being able to closely emulate the RCT [26,27,28]. The use of causal inference frameworks in RWD studies has not been routinely incorporated into RWD protocols, but it will improve the robustness of conclusions from observational studies that produce RWE. The credibility of RWD for causal inference from observational studies has been a matter of debate; these studies must be carefully designed, and statistical methods must be applied to address the potential for bias and confounding [29]. The use of RWD for causal inference has been advanced by the work of Hernan and colleagues, who advocate for a target trial simulation approach to study design, which also has implications for how we think about data quality. This approach enables a qualitative assessment of how closely RWD studies can emulate the theoretical RCT that one would perform, if feasible.

Recent advances in statistical methods for causal inference in epidemiology (e.g., doubly robust methods and G estimation) are all based on estimating treatment effects using the mean difference in predicted outcomes for the intervention and comparison groups rather than estimating a parameter in an equation [30,31]. These methods may enable a quantitative assessment of residual bias and confounding. Because the treatment effects are based on predictions, machine learning methods are particularly useful for implementing these new statistical estimates of treatment effects. RWD needs to be adequate for estimating good predictions, but it is not clear that we need equally high levels of data quality for all the variables in predictive models.

## 4. System Interoperability and Data Privacy

It has become clear that the value of RWD increases as various data sources are linked together. Accurate linkage of different data sources requires overcoming the challenges of interoperability. Interoperability, particularly in the US, where data are often siloed. In most instances, common data models (CDMs) are employed, which organize data into a standard structure and apply standard data definitions [32,33]. These typically vary across networks. Standardizing data curation across these disparate data types and sources presents formidable challenges, requiring consensus among stakeholders and alignment with industry best practices.

Technical challenges include different systems and linkage issues such as non-unique patient identification numbers, semantics, and various CDMs used. This has frequently been addressed using probabilistic linkage techniques. As discussed earlier, recent efforts to address this involve data tokenization; for example, the standards are established under Health Level Seven International (HL7) and Integrating the Healthcare Enterprise (IHE) [34,35].

Initiatives such as EHR adoption, interoperability standards, and data exchange platforms aim to streamline data sharing and integration, enabling stakeholders to access and analyze data more efficiently. Despite these advancements, siloed data remains a significant barrier to realizing the full potential of RWD in the US.

In contrast, the EU landscape for RWD is characterized by initiatives aimed at facilitating data discovery and exchange. The EHDS seeks to foster cross-border data exchanges to support healthcare delivery, research, and innovation. EHDS aims to overcome barriers to data sharing while ensuring compliance with privacy regulations, thereby enabling stakeholders to access a wealth of RWD from diverse sources across member states.

Additionally, metadata catalogs play a crucial role in facilitating data discovery within the EU. These catalogs provide comprehensive information about the available datasets, including data types, sources, and access requirements, enabling researchers to identify relevant datasets for their specific research questions. However, challenges persist due to varying levels of digitalization across member states, as well as differences in data governance frameworks and privacy regulations.

Data privacy considerations loom large when leveraging RWD, particularly given the sensitive nature of health data. Privacy concerns are addressed through regulatory frameworks such as the Health Insurance Portability and Accountability Act (HIPAA) and General Data Protection Regulation (GDPR) in the US and EU, respectively [36,37]. Challenges remain to ensure compliance and safeguard patient data. Safeguarding patient privacy and ensuring compliance with regulatory requirements are critical considerations in the collection, use, and sharing of RWD. Stringent privacy protections, such as de-identification techniques, encryption protocols, and access controls, are essential for mitigating the risks of unauthorized access, data breaches, and privacy violations. By prioritizing patient privacy and data security, stakeholders can foster trust and confidence in RWD/RWE initiatives, enabling data-driven decision-making while respecting individual privacy rights.

The foremost among these was HIPAA, enacted in 1996, which establishes national standards for the protection of sensitive patient health information. HIPAA mandates stringent safeguards for the handling and transmission of health data, imposing penalties for non-compliance.

In addition to HIPAA, recent legislative developments in the US include the California Consumer Privacy Act (CCPA) [38]. The CCPA enhances consumer privacy rights and imposes obligations on businesses regarding the collection, use, and sale of personal information. Although not specifically tailored to healthcare data, the CCPA has implications for RWD initiatives, particularly in the context of patient privacy and data sharing. The California Privacy Rights Act (CPRA) was passed, and the CPRA amended and extended the CCPA [39].

Both HIPAA and GDPR play a crucial role in safeguarding patient privacy and establishing standards for data protection in the US and EU, respectively. However, challenges remain in ensuring compliance and safeguarding patient data in the face of evolving threats and technological advancements. Healthcare organizations must remain vigilant and proactive in implementing robust security measures to protect patient data and uphold high standards of privacy and confidentiality.

## 5. Discussion

The importance of RWD and RWE is ever-increasing and is foundational to the creation of a learning healthcare system. From optimizing treatment pathways and identifying real-world safety concerns to informing regulatory decisions and shaping healthcare policy, applications using RWD for RWE can be vast and far-reaching. Through data discoverability, ensuring quality, protecting privacy, and promoting interoperability, RWD and RWE initiatives enable stakeholders to harness the power of big data and evidence to drive positive change and innovation in healthcare.

In a learning healthcare system, new insights are not only learned from discrete experiments, such as standard CTs, but also from a range of investigations that utilize RWD. The range includes randomized interventional studies (e.g., PCTs) to non-randomized interventional studies (e.g., single-arm studies utilizing an external control group) to non-randomized non-interventional studies or observational studies (e.g., prospective observational studies and registries) [40].

As healthcare continues to evolve over time, the richness of RWD will continue to expand and improve in quality. By appropriately harnessing the diverse forms of RWD as they become available, stakeholders can unlock new opportunities for improving patient care, advancing research, and informing policies and decision-making in healthcare. This will be augmented by additional advances in the study designs and statistical analyses. Advances in artificial intelligence (AI) may be a core part of this era of big data [41,42,43]. However, validation of AI on a use-case basis is required to ensure that their findings are applicable and beneficial to larger, diverse patient populations. This is especially true for regulatory decision-making.

## 6. Conclusions and Future Direction

RWD and RWE applications will increase in the future, given the abundance of data from various sources. Standard RCTs alone will not adequately address the complex intersection of many diseases and comorbid conditions, which are patient-centric and require us to find alternate ways of getting evidence to support such gaps. Big data, RWD, digital, AI, and robotics have the potential to support patients for generalizability across the spectrum of various characteristics and comorbid conditions while considering the tradeoff between potential benefits and risks, as well as the data privacy rules.

Concerns about equity and diversity will still loom large. Biases in data collection, algorithmic bias, and disparities in access to healthcare resources can exacerbate inequities and perpetuate systemic biases in healthcare delivery and outcomes. Addressing these concerns will require a concerted effort to ensure inclusivity, fairness, and transparency in development.

The deployment of AI-driven healthcare solutions must be executed in a deliberate fashion, accompanied by transparency, explicability, and use validation. By prioritizing equity and diversity, stakeholders will need to mitigate biases, promote health equity, and ensure that data and technologies can benefit all patient groups. It is anticipated that RWD and RWE will play increasing roles in the biopharma industry as issues of ethics, transparency, and trustworthiness are addressed.

Actions by regulatory agencies and HTA authorities will have a significant impact on the speed and application of RWE in population-based decision-making. As noted earlier, there is ample room for the harmonization of data quality frameworks. In addition, how the specific consideration of causal inference is incorporated into the study design and analysis is in its early stages. The adoption of international standards for “good study practices” also needs to be addressed for non-interventional studies. The latter has been less of an issue for descriptive RWE studies (e.g., treatment patterns, disease progression) and for safety studies; however, they will be critical for studies of treatment effectiveness. See, for example, best practices [44]. Achieving consensus on data quality frameworks, study design, and analysis standards will likely have a greater short-term impact on the adoption of RWE than the creation of new RWD sources.

## Data Availability

Not applicable.
